# Editorial: Effects of Early Life Stress on Neurodevelopment and Health: Bridging the Gap Between Human Clinical Studies and Animal Models

**DOI:** 10.3389/fnhum.2021.751102

**Published:** 2021-10-05

**Authors:** Arie Kaffman, Ryan J. Herringa, Mar M. Sanchez

**Affiliations:** ^1^Department of Psychiatry, Yale University School of Medicine, New Haven, CT, United States; ^2^Department of Psychiatry, University of Wisconsin School of Medicine and Public Health, Madison, WI, United States; ^3^Department of Psychiatry & Behavioral Sciences, School of Medicine, Emory University, Atlanta, GA, United States; ^4^Division of Developmental and Cognitive Neuroscience, Yerkes National Primate Research Center, Emory University, Atlanta, GA, United States

**Keywords:** early life adversity, early life stress, translational research, health, animal models, peripheral biomarkers

Early life adversity (ELA) is a broad term used to describe environmental conditions that negatively impact normal development (Shonkoff et al., 2012). These include different forms of parental maltreatment/neglect, bullying, poverty, exposure to war or neighborhood crimes, discrimination, malnutrition, and environmental pollution. In many cases, these toxic environmental conditions co-occur leading to impairment in normal development and the emergence of multiple psychiatric and other medical conditions (Nemeroff, 2016; Teicher and Samson, 2016). A recent analysis estimated the economic burden of childhood maltreatment in the US alone at 2 trillion dollars per year (Peterson et al., 2018), far exceeding estimated costs of all cancers combined (Mariotto et al., 2011). Precisely how ELA alters neurodevelopment and increases the risk for such a broad range of psychiatric and medical conditions and how can these effects be reversed are difficult questions to address in humans. This is due to the heterogeneity of the adversities, differences in genetic susceptibility, and limited tools to rigorously examine and manipulate the human brain especially early in development (Smith and Pollak, 2021). Animals exposed to ELA show several structural and behavioral changes reported in humans and this preclinical work has provided some important insights into possible mechanisms and novel diagnostic and therapeutic interventions (White and Kaffman, 2019). Unfortunately, much of the clinical and preclinical efforts run in parallel tracts with little coordination and cross talk between the two approaches.

The goal of this eBook was to solicit input from researchers on both sides of the “scientific divide” on how to better coordinate the effort between clinical and preclinical/basic neuroscience and to highlight specific areas that show great promise for a productive translational collaboration and the development of new diagnostic and therapeutic tools. The response from the scientific community on how to bridge the gap between pre-clinical and clinical work was both fascinating and diverse and we conceptually grouped it into 5 themes: (1) Novel Mechanisms and Potential Therapies, (2) Insights Regarding Prenatal Stress, (3) Cumulative vs. Dimensional Models, (4) The Moderating Effects of Sex, and (5) Gene by Environment Interaction.

## Novel Mechanisms and Potential Therapies

Several papers provided novel mechanistic insights that may lead to the development of new screening tools and interventions. Islam and Kaffman point out that ELA perturbs myelin development in humans, non-human primates and rodents. The authors review novel mechanistic insights from recent studies in rodents and discuss specific examples of how insights from animal studies coupled with the development of novel myelinating agents provide a promising new pharmacological frontier to treat cognitive and behavioral consequences of ELA in humans. Guadagno et al. review recent findings showing that some forms of adversities disrupt the development of perineural nets surrounding GABAergic neurons in rodents and humans. They propose that disruption of these structures located around neurons alters inhibitory/excitatory outputs in fronto-limbic circuits that are responsible for threat detection and anxiety. This is a novel and intriguing mechanistic animal model nicely complements Hanson and Nacewicz proposal that disruption of inhibitory/excitatory balance drives ELA mediated changes in amygdala volume (see “Cumulative vs. Dimensional Models” section below).

Coplan et al. report that the kynurenine pathway is dysregulated in the brain of adult macaque females exposed to variable foraging demand -a form of ELA- as infants, with concentrations of kynurenine metabolites in cerebrospinal fluid (CSF) serving as good markers of the different routes of the pathway. This is the first study to describe changes in kynurenine metabolites in an animal model of ELA, providing a novel mechanism and a potential therapeutic target to address neuroimmune modulation seen in some early adversities. Duffy and Roth provide a thoughtful discussion about the challenges of using peripheral epigenetic markers of DNA methylation to predict changes in the prefrontal cortex using a maternal-maltreatment rat model.

## Insights Regarding Prenatal Stress

Several authors focused on prenatal stress as an important area of productive translational work. Duffy et al. reviewed elegant mechanistic studies in rodents indicating that prolonged parental stress alters non-coding small RNA content in the sperm that in turn guides differences in neurodevelopment, physiology, and behavior in the offspring. These preclinical findings provide a novel conceptual framework for evaluating similar changes in non-coding small RNAs sperm content seen in stressed men. Fitzgerald et al. highlights the chronicity of prenatal stress in clinical settings as opposed to the more acute stress used in rodent models of prenatal stress. The authors explore differences in outcomes between chronic and acute prenatal stress and advocate for the inclusion of more chronic prenatal stress paradigms in rodents.

Perinatal iron deficiency is associated with abnormal neurodevelopment and is commonly seen in early adversities associated with severe poverty and famine. Using a naturalistic non-human primate model of prenatal iron deficiency, Vlasova et al. found structural MRI changes in both gray and white matter of 1 year old infants diagnosed with iron deficiency during the perinatal period (i.e., 1–2 months). Interestingly, repleting iron stores during the perinatal period did not reverse the MRI changes suggesting that earlier preemptive supplementation of iron, especially during the prenatal period, might be necessary.

Ceniceros et al. present surprising and thought-provoking results in macaques suggesting that maternal relocation during pregnancy reduced measures of anxiety in 3–4 month old offspring. These findings were also seen in infants that were cross-fostered postnatally and are consistent with the notion that mild-stressors during the prenatal period might have an inoculative/protective effect on neophobia in the offspring. Finally, Georg et al. used machine learning to identify variables that increase parental stress in response to infants with early regulatory disorder (i.e., colic). The authors suggest that interventions that reduce parental stress will likely improve long-term outcomes in infants with regulatory disorder.

## Cumulative vs. Dimensional Models

Several articles provided interesting insights into a central debate between the “cumulative risk model” in which multiple stressors are simply added to predict health outcomes (Evans et al., 2013) and the “multi-dimensional model” arguing that distinct types of adversities cause different developmental outcomes that are not necessarily additive and are better characterized using a multi-dimensional grid (Mclaughlin et al., 2014; Mclaughlin and Sheridan, 2016). Using animal and human studies, Hanson and Nacewicz propose an inverted-U-allostatic load model as a conceptual framework to explain inconsistent findings on the effects of ELA on amygdala size. Simply put, low-moderate levels of adversity lead to relative amygdala expansion whereas sustained and severe activation of the amygdala during childhood and adolescence leads to excitatory neurotoxic-mediated dendritic atrophy, possibly cell death, and a reduction in amygdala volume. The authors propose that physical or mental perception of “entrapment/inescapability” is a critical dimension driving changes in excitatory/inhibitory balance and ultimately amygdala volume.

Gotlib et al. reported that severity of ELA predicted levels of depression during the covid pandemic in adolescents. This severity effect was more pronounced in females and was mediated by perceived levels of stress. These findings highlight the role that “perceived stress” plays in mediating the risk for depression and suggest that clinical interventions that target stress perception may be particularly effective in individuals exposed to high levels of adversities. It is tempting to speculate that levels of “perceived stress” noted by Gotlib et al. is highly related to the perception of “entrapment” emphasized by Hanson and Nacewicz.

Using a rat model of ELA, Zhang et al. reports an interesting relationship between levels of BDNF in the prefrontal cortex of juvenile rats and the severity of the adversity. More specifically, juvenile rats exposed to 3 h of maternal separation had lower BDNF in the prefrontal cortex, and had higher levels of anxiety and corticosterone compared to juvenile rats exposed to only 15 min of maternal separation. These preclinical findings raise the possibility that levels of BDNF in the prefrontal cortex may partly mediate the cumulative risk outcomes seen clinically across multiple psychiatric conditions. Orso et al. reports robust increase in anxiety-like behavior in mice exposed to a combination of two forms of early adversities (i.e., maternal separation and limited bedding and nesting). These findings underscore the need for additional preclinical work studying the effects of “simple,” “multiple,” and “co-occuring” stressors on neurodevelopment. Such work will better mimic clinical settings and help address the different predictions made by the cumulative and the dimensional models (White and Kaffman, 2019).

## The Moderating Effects of Sex

The notion that males and females respond differently to ELA has received growing attention in recent years including some very interesting findings reported in this collection. For example, consistent with previous work, Stenson et al. found that ELA accelerated puberty in black girls. Using statistical mediation analysis they also showed that accelerated puberty significantly contributed to increased anxiety in black girls. ELA did not accelerate puberty in black boys and there was no significant relationship between timing of puberty and anxiety, highlighting important differences in the effects of ELA on puberty and anxiety in males and females. Raymond et al. found that women that were exposed to ELA after age 8, but not before age 8, showed increased attentional bias to threat, findings that were not seen in men. Clarifying the underlying mechanisms by which sex alters outcomes of ELA is an area of preclinical research that desperately needs additional attention due to the long standing historical bias of conducting animal studies exclusively in males (White and Kaffman, 2019; Bath, 2020).

Ultrasonic vocalizations (USVs) in rodent pups is the equivalent of an infant cry and an effective mechanism to solicit maternal care. Granata et al. showed that exposure to daily maternal separation altered USVs mainly in males, while exposure to limited bedding and nesting (a different model of ELA) impacted USVs mainly in females indicating that males and females are affected differently by different forms of adversities. An interesting follow up question is whether exposing pups to both stressors simultaneously, as was done by Orso et al. would impact both sexes and whether the magnitude of the changes would be enhanced in the presence of this complex stress.

## Gene by Environment Interaction

The moderating effects of genetic susceptibility on consequences of ELA have been re-examined in this eBook using two well characterized genes: FKBP and the serotonin transporter. The FKBP gene plays an important role in terminating glucocorticoid mediated stress response and has been previously shown to interact with ELA to alter risk of depression and PTSD (White et al., 2012; Klengel et al., 2013). Kwon et al. extended these earlier findings to show that susceptible alleles of the FKBP gene interact with ELA to modulate volumetric changes in the left orbitofrontal cortex and the left middle temporal gyrus in non-symptomatic individuals. Wood et al. examined the effects of peer-rearing and the serotonin transporter alleles on baseline and stress-induced monoamine levels in a large cohort of infant macaques. Infants that were raised with peers in the absence of a mother had different baseline and stress-induced monoamine levels compared to infants raised by their mother in a more complex and naturalistic social context. This work also provides new insights into earlier findings in humans showing that individuals the “short” allele are more sensitive to ELA compared to individuals with the “long” allele (Caspi et al., 2003).

## Author Contributions

AK wrote the first draft which was then edited and modified by RH and MS. All authors contributed to the article and approved the submitted version.

## Funding

This work was supported by: NIMH R01MH118332 (AK), NIMH R01MH119164 (AK), NIMH R01MH117141 (RH), R01MH115910 (RH), NIDA DA038588 (MS), NIDA R01 DA052909, NICHD HD077623 (MS), and NIH's Office of the Director, Office of Research Infrastructure Programs, P51OD011132 (Yerkes National Primate Research Center Base grant).

## Conflict of Interest

The authors declare that the research was conducted in the absence of any commercial or financial relationships that could be construed as a potential conflict of interest.

## Publisher's Note

All claims expressed in this article are solely those of the authors and do not necessarily represent those of their affiliated organizations, or those of the publisher, the editors and the reviewers. Any product that may be evaluated in this article, or claim that may be made by its manufacturer, is not guaranteed or endorsed by the publisher.
